# Reproductive toxicity of Momordica charantia ethanol seed extracts in male rats

**Published:** 2014-10

**Authors:** Panas Tumkiratiwong, Ravicha Ploypattarapinyo, Urai Pongchairerk, Wachiryah Thong-asa

**Affiliations:** 1 *Physiology Division, Department of Zoology, Faculty of Sciences, Kasetsart University, Bangkok, 10900, Thailand. *; 2 *Department of Anatomy, Faculty of Veterinary Medicine, Kasetsart University, Bangkok, 10900, Thailand.*

**Keywords:** *Infertility*, *Momordicacharantia*, *Reproduction*, *Testosterone*, *Toxicity*

## Abstract

**Background:** *Momordica charantia* (*M. charantia*) seed has been supposed to have an antifertility property but mechanisms underlying the infertility effect have not been investigated.

**Objective:** We investigated the antifertility effect of *M. charantia* ethanol seed extracts on reproductive toxicology and seminal and plasma testosterone in male Wistar rats.

**Materials and Methods: **The control group (I) was provided daily 1 ml dimethylsulfoxide (DMSO) and the experimental groups II and III were given daily 400 and 800 mg dry matter/kg body weight of the extracts dissolved in 1 ml DMSO via the esophageal route. All groups were administered for 42 days (day 42). Changes in body weight, fertility, reproductive characteristics, testicular histopathology and levels of seminal and plasma testosterone among three groups were compared.

**Results: **On day 42, the extracts caused antifertility (p=0.001). The extracts demonstrated significant reductions in diameters of seminiferous tubules and epididymides, spermatid density, daily sperm production and caudal epididymal spermatozoa, sperm motility and viability (p=0.046). Pathological changes in seminiferous tubules revealed atrophy, desquamation, pyknosis nucleus and multinucleated giant cell. Plasma cells were evident in three parts of epididymides of rats treated with high dose of the extract. Furthermore, the high dose of the extract suppressed seminal testosterone level (p=0.001) and plasma testosterone level (p=0.002).

**Conclusion:** Our data showed that high dose of *M. Charantia* seed extracts caused infertility in male rats. The interruption in their fertility was probably attributed to the direct toxic to seminiferous tubules, epididymis and the lowered testosterone level which might impact on sperm parameters.

## Introduction

Some herbal extracts have been proven to have effects on male infertility, for example, gossypol, papaya seed and neem oil and neem seed (1, 2). We are interested in mechanisms underlying the male infertility of *Momordica charantia *(*M. charantia*) seed which its chemical constituents are β-sitosterol-β-D-glucoside; stearic acid; lectins; two triterpene glycosides: momordicosides A and B; momordicosides C, D, and E; two cytokinins: zeatin and zeatin riboside; two proteins: α- and β-momorcharins; p-cymene; hexadecanol; menthol; nerolidol; pentadecanol; squalene; 10α-cucurbit-5,24-dien-3β-ol; 24-methylen-cycloartanol; taraxerol; β-amyrin; campesterol; cycloeucalenol; 24β-ethyl-5α-cholesta-7-trans-22-dien-3β-ol; 24β-ethyl-5α-cholesta-7-trans-22,25-trien-3β-ol; lophenol; 4α-methylzymosterol; obtusifoliol; spinasterol; stigmasterol; stigmasta-7,25-dienol; stigmasta-7,22,25-trienol; momordica anti-protein (MAP 30); and ribosome-inactivating-proteins (RIPs) (3, 4). *M. charantia* ethanol seed extracts at 25 mg/100 g body weight can effect spermatogenic process in Albino rats and in mice (5, 6). *M. charantia* methanolic seed extracts at 50 mg/100 g body weight caused in male Sprague‐Dawley rats infertility (7). *M. charantia* seeds also induced abortions in Albino rats (6). 

Sharanabasappa *et al* reported that *M. charantia* seed extracts given to female Albino rats at a dose level of 25 mg/100 g body weight for 30 days have antiovulatory activities such as a reduced ovarian weight, a decreased number of developing follicles, Graafian follicles, and corpora lutea, and an increased number of atretic follicles (8). The other mechanisms underlying the male infertility of *M. charantia* seed extracts have not been investigated. We, therefore, investigated the antifertility effect of *M. charantia* ethanol seed extracts on reproductive toxicology and seminal and plasma testosterone in male Wistar rats.

## Materials and methods

**Plant materials and preparation of ethanolic extract of** ***M. charantia***

Fresh raw *M. charantia* were collected. The white arils were discarded and then the seeds were collected separately, air dried in shadow, powdered, and extracted with 70% ethanol (v/v) using a soxhlet apparatus. The extracts were evaporated, dried under vacuum, and kept at 4^o^C for further usage. A suspension of *M. charantia* ethanol seed extracts was prepared in 1 ml dimethylsulfoxide (DMSO) prior to daily administration via the esophageal route for consecutive 42 days (day 42).

***Animals***

Male Wistar rats, aged 9 weeks and weighing 300 g± 10 g, were introduced from the National Laboratory Animal Center (NLAC), Mahidol University, and were allowed to be acclimatized to our laboratory for 7 days prior to the treatment. All rats were individually housed in stainless steel metabolic cages (20× 30× 13 cm). The controlled temperature ranged from 22-24^o^C with relative humidity ranging from 55-60% and a daily cycle of 12 hr light and 12 hr darkness. We conducted this experiment in the departmental animal house at Physiology Division, Department of Zoology, Faculty of Science, Kasetsart University.

They were provided with a commercial diet and water ad libitum. Male rats were selected for fertility testing prior to be employed in the experiment. The Animal Ethics Committee of Kasetsart University has approved the experimental protocol according to the ID # ACKU 01052.

**Experimental design**

Fertile-proven male Wistar rats were allocated according to their body weight into three groups of 14 animals in each group: in group I, each animal was given DMSO 1 ml per day as a vehicle; in group II, *M*.* charantia* ethanol seed extracts at 400 mg dry matter/kg body weight suspended in 1 ml DMSO was provided daily to each animal; in group III, *M*.* charantia* ethanol seed extracts at 800 mg dry matter/kg body weight suspended in 1 ml DMSO was given daily to each animal. In this experiment, the doses of *M. charantia* ethanol seed extracts at 400 and 800 mg dry matter/kg body weight were introduced according to Abalaka *et al* who reported that LD_50_ of edible parts of *M. charantia* extracts that caused an acute toxicity was 1200 mg dry matter/kg body weight (9).

One half of animals in each group (7 animals) were remained for consecutive 14 days without treatment as a recovery period (day 56). On day 42 (7 animals/group) and day 56 (7 animals/group), all male Wistar rats were sacrificed under light ether and then testes were removed and cleared of blood vessels and clots by rinsing with a phosphate buffered saline (PBS) solution, pH 7.4. The testes were then used for further monitor various reproductive characteristics, seminiferous tubule and epididymal histopathology, and seminal testosterone levels.

**Fertility testing**

Female rats in proestrous phase were introduced to males in a ratio of 2:1. Females that had sperm plugs from the vaginal smear were separated, and pregnancy was maintained for 10 days. Mated females were then sacrificed with light ether, and male fertility index was tested by counting the number of foetuses divided by the number of corpora lutea (10). Each male with fertility equal to or greater than 85% was chosen for further random allocation into three groups according to their body weight. Fertility was also evaluated on day 42 (42 days of *M. charantia* ethanol seed extracts treatment) and day 56 (14 days of *M. charantia* ethanol seed extracts withdrawal).

**Determination of testicular and germ cell characteristics**

Reproductive characteristics were monitored, namely reproductive organ weight: testis and epididymis, prostate gland, seminal vesicle; microscopic diameter of seminiferous tubule and of caput, corpus and caudal parts of epididymis. Germ cell characteristics: the number of spermatogonia, spermatocytes and spermatids (cells/1000 μm^2^); spermatozoa quantity: daily sperm production and caudal epididymal spermatozoa; spermatozoa quality: sperm motility and viability, and normal spermatozoa; types of abnormal spermatozoa (200 cells): tailless, no hook head, amorphous head, neck and tail abnormal; acrosomal intactness; and acrosome-membrane intactness: acrosome-membrane intact, acrosome damage-membrane intact, acrosome intact-membrane damage, and acrosome-membrane damage (10, 11).

**Histopathology**

The testis; and caput, corpus and caudal parts of epididymis were excised and fixed in a 10% v/v buffered neutral formalin solution, processed by the paraffin technique (12). The tissue was cut in cross-section to 5 µm in thickness using a LEICA RM2145, (Heidelberger str. 17-19, D-69226 Nussloch Germany). Sections were stained with hematoxylin and eosin and then observed under a light microscope (12). The seminiferous tubules were evaluated for the existence of complete spermatogenesis and for atrophy, desquamation, pyknosis nucleus and multinucleated giant cells. The alterations of epididymis were also investigated on the presence of plasma cell and multinucleated giant cell (13).

**The determination of seminal and plasma testosterone levels**

The preparation of seminal fluid for determination of testosterone level was followed by the modified method of Abul *et al* (14). Prior to testosterone determination, seminal fluid was mixed with STE buffer in ratio of 1:9 but plasma was not diluted with STE buffer. The seminal and plasma levels of testosterone were measured by radioisotope ^125^I radioimmunoassay (RIA) by using a Coat A Count^®^ Testosterone Kit (Diagnostic Products, Los Angeles, CA) radioimmunoassay. 

The intra-assay and inter-assay variation expressed as coefficient of variation (%CV) were 6.1 and 9.3%, respectively. The approximate sensitivity of this assay was 0.017 ng/ml. The percent cross-reactivity with androstenedione and androstenediol was 1.6 and lower than 0.1%, respectively. The spiking recovery values averaged 94.42%. The parallelism of 8600 ng/ml (undiluted concentration) with 50.0, 25.0 and 12.5% dilution of its diluted concentrations were 4350, 2350, 1005 ng/ml, respectively. Seminal and plasma testosterone concentrations were calculated by using GMS Version 3.05: GAMMA-C12 to produce the standard curve of calibrators and logit-log graph of samples.

**Body weight measurement**

Body weight of all fertile-proven male Wistar rats was measured weekly to adjust the dosage of the extracts according to body weight of individuals and also to monitor changes in their body weight throughout the experiment.

**Statistical analysis**

Data were expressed as the mean±SD. Normal distribution and homogeneity of variances were analyzed employing Kolmogorov-Smirnov’s test and Levene’s test, respectively. One-way analysis of variance (ANOVA) was used and the mean differences among groups were analyzed by least significant difference (LSD). The threshold of significance was set to p<0.05 for all parameters.

## Results

**Changes in body weight**

**Figure 1:**
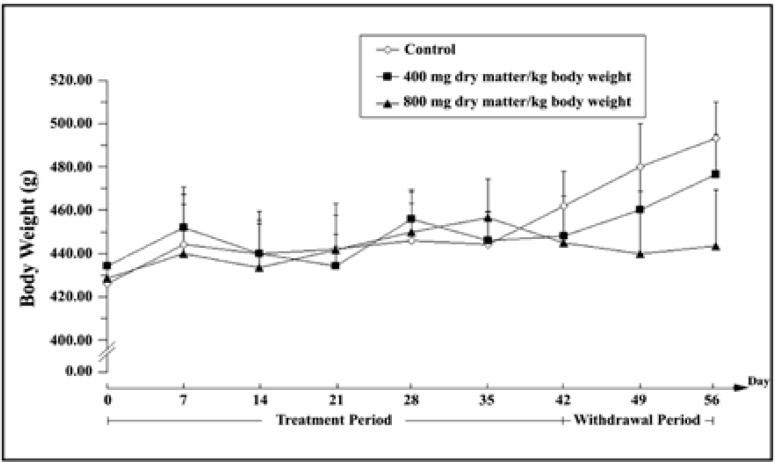
Weekly changes in body weight of male Wistar rats of three groups prior to (day 0), treatment period (day 42) and withdrawal period (day 56). Results were expressed as the mean±SD (n=7 per group). Values not shown by any letter were not significant difference at p<0.05

This study demonstrated that changes in body weight had no significant difference among three groups during 42 days of treatment (day 42) and 14 days of the withdrawal (day 56) periods (p>0.05) (Figure 1).

**Male fertility**

**Figure 2:**
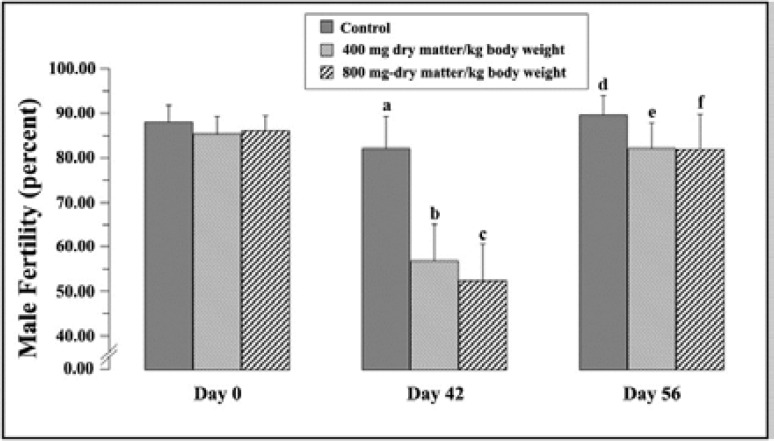
Male fertility percentage of Wistar rats on day 0, 42 and 56 of the experiment. Each point represented the mean±SD (n=7 per group). p<0.05 showed significant differences (^a, b^ p=0.001; ^a, c^ p=0.001; ^b,c ^p=0.366; ^d, e ^p=0.347; ^d, f^ p=0.347; ^e, f^ p=0.987).

There was no significant difference in fertility among the three groups on day 0 (p=0.671) but significant infertility on day 42 was observed in Wistar rat treated with *M. charantia* ethanol seed extracts at 400 and 800 mg dry matter/kg body weight (p=0.001; 0.001, respectively) as compared to the control group. Following 14 days of the withdrawal of the extracts, infertility was restored to normal and not significant to the control group (p=0.347; 0.347, respectively) (Figure 2).

**Reproductive characteristics**

This study showed that on day 42, the extracts at 400 and 800 mg dry matter/kg body weight insignificantly reduced testicular, epididymal, prostate gland and seminal vesicle weight among three groups (p>0.05) (Table I). The extracts significantly reduced the diameter of seminiferous tubules and of epididymides as compared to the controlled group (p<0.05) (Table I). The number of spermatogonia and spermatocytes were not significant difference in Wistar rats treated with the extracts as compared to the controlled group (p>0.05) (Table I). There were significant reductions in percent of spermatozoal motility and viability, and normal spermatozoa in Wistar rats treated with the extracts as compared to the controlled group (p<0.05) (Table I). The motility of spermatozoa was completely established in epididymis. Therefore, damages to caput, corpus and caudal parts of the epididymis imparted an efficacy of sperm motility. The tailless, no hook head, amorphous head, and neck and tail abnormal types of spermatozoa tended to be insignificantly lowered as compared to the controlled group (p>0.05) (Table I). 

The percentage of acrosomal intactness was not significant difference among three groups (p>0.05) (Table I). There was trend to be insignificantly lowered in acrosome-membrane intact as compared to the controlled group (p>0.05) (Table I). The percentages of acrosome damage-membrane intact and acrosome-membrane damage tended to be insignificantly increased as compared to the controlled group (p>0.05) (Table I). The percentage of acrosome intact-membrane damage was not significant difference among three groups (p>0.05) (Table I). In this study, following the withdrawal of the extracts, all parameters mentioned above were restored to normal (p>0.05) (Table I).

**Seminiferous tubule and epididymal histopathology**

**Figure 3:**
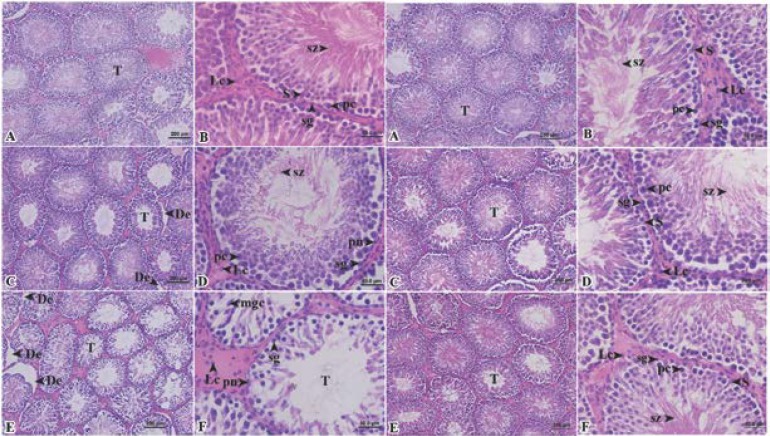
Column 1 and 2: photomicrographs of seminiferous tubules on day 42; Column 3 and 4: photomicrographs of seminiferous tubules on day 56: A and B, group I, control group; C and D, group II, 400 mg dry matter/kg body weight; and E and F, group III, 800 mg dry matter/kg body weight (De, desquamation; Lc, Leydig’s cell; mgc, multinucleated giant cells; pc, primary spermatocyte; pn, pyknosis nucleus; S, Sertoli cell; sg, spermatogonia; sz, spermatozoa and T, tubular atrophy).

More and much more atrophies of seminiferous tubules were found in Wistar rats treated with the extracts at 400 and 800 mg dry matter/kg body weight (Figure 3C and 3E: column 1). 

Desquamation and pyknosis nucleus were appeared in seminiferous tubules of Wistar rats treated with the extracts (Figure 3C and 3E: column 1; 3D and 3F: column 2, respectively) as compared to the controlled group (Figure 3A and 3B: column 1 and 2). In seminiferous tubules of Wistar rats treated with the extracts at 800 mg dry matter/kg body weight, the small number of spermatids and a multinucleated giant cell were observed (Figure 3F: column 2). On the withdrawal of the extracts, all parameters mentioned above were restored to normal (Figure 3C and 3E: column 3; 3D and 3F: column 4).

**Figure 4:**
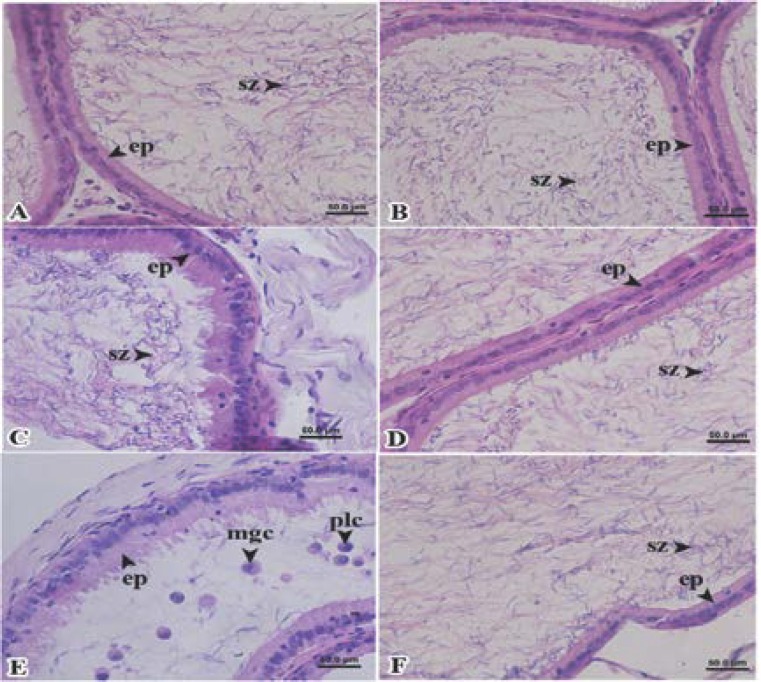
Column 1: photomicrographs of caput of epididymis on day 42 and column 2: on day 56: A and B, group I, control group; C and D, group II, 400 mg dry matter/kg body weight and E and F, group III, 800 mg dry matter/kg body weight. (ep, epithelium cell; mgc, multinucleated giant cells; plc, plasma cell and sz, spermatozoa).

We found more plasma cells and more multinucleated giant cells in caput part of epididymis of Wistar rats treated with the extracts at only 800 mg dry matter/kg body weight (Figure 4E) as compared to the control group (Figure 4A) and the group treated with the extracts at 400 mg dry matter/kg body weight (Figure 4C). The less number of spermatozoa were appeared in caput part of epididymis of Wistar rats treated with the extracts, especially at 800 mg dry matter/kg body weight (Figure 4E). Following the withdrawal of the extracts, there was no appearance of plasma cell and multinucleated giant cell (Figure 4F).

**Figure 5:**
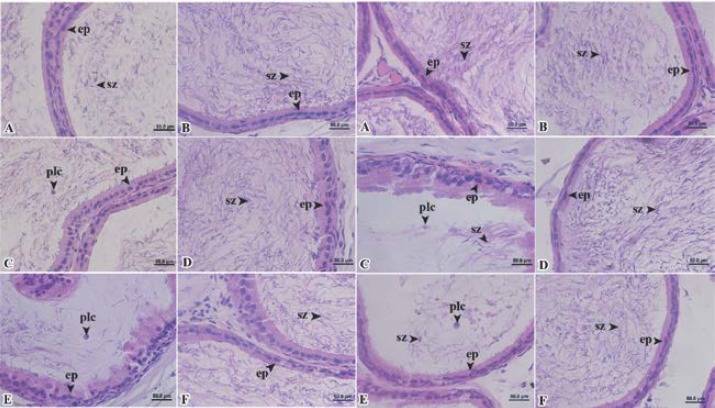
Column 1: photomicrographs of corpus of epididymis on day 42, column 2: of corpus of epididymis on day 56, column 3: photomicrographs of cauda of epididymis on day 42 and column 4: of cauda of epididymis on day 56: A and B, group I, control group; C and D, group II, 400 mg dry matter/kg body weight and E and F, group III, 800 mg dry matter/kg body weight (ep, epithelium cell; plc, plasma cell and sz, spermatozoa).

Plasma cells in the corpus and caudal parts of epididymis were found in Wistar rats treated with the extracts (Figure 5C and 5E: column 1 and 3) as compared to the control group (Figure 5A: column 1 and 3). The few number of spermatozoa were also appeared in the corpus and caudal parts of epididymis of Wistar rats treated with the extracts (Figure 5C and 5E: column 1 and 3). On the withdrawal of the extracts, all parameters mentioned above were restored to normal (Figure 5D and 5F: column 2 and 4) as compared to the control group (Figure 5B: column 2 and 4).

**Seminal and plasma testosterone levels**

**Figure 6:**
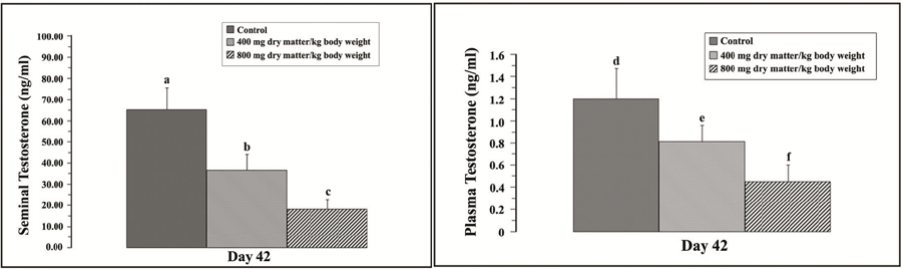
Seminal testosterone (left) and plasma testosterone (right) levels on day 42. Each point represented the mean±SD (n=7 per group). p<0.05 showed significant differences (^a, b^ p=0.021; ^a, c^ p=0.001; ^b,c ^p=0.122; ^d, e ^p=0.036; ^d, f^ p=0.002; ^e, f^ p=0.156).

The extracts at 400 and 800 mg dry matter/kg body weight lowered the seminal testosterone level (p=0.021; 0.001, respectively) as compared to the control group (Figure 6, left) and plasma testosterone levels (p=0.036; 0.002, respectively) as compared to the control group (Figure 6, right).

**Table I:** Testicular and diverse germ cell characteristics

**Testicular and germ cell characteristics**		**Day 42 (N=7 rats/group)**			**Day 56 (N=7 rats/group)**		
		**Control**	**400 mg dry matter/kg body weight/day**	**800 mg dry matter/kg body weight/day**	**Control**	**400 mg dry matter/kg body weight/day**	**800 mg dry matter/kg body weight/day**
Reproductive organ weight (g/100 g body weight)							
	testis	0.48 ± 0.03	0.43 ± 0.04	0.42 ± 0.04	0.39 ± 0.01	0.40 ± 0.01	0.42±0.01
	epididymis	0.12 ± 0.01	0.12 ± 0.00	0.12 ± 0.01	0.11 ± 0.00	0.11 ± 0.00	0.11 ± 0.01
	prostate gland	0.10 ± 0.01	0.09 ± 0.01	0.09 ± 0.01	0.09 ± 0.02	0.08 ± 0.00	0.10 ± 0.02
	seminal vesicle	0.14 ± 0.00	0.13 ± 0.01	0.13 ± 0.00	0.13 ± 0.01	0.13 ± 0.01	0.12 ± 0.01
	Seminiferous tubule diameter (µm)	211.50 ± 1.63^a^	199.11 ± 1.44^b^	173.13 ± 1.59^c^	217.65 ± 1.29	220.57 ± 1.39	217.38 ± 1.36
Epididymal diameter (µm)							
	caput part	261.72 ± 5.76^a^	238.21 ± 5.65^b^	173.84 ± 2.95^c^	269.82 ± 4.00	257.87 ± 4.34	263.93 ± 4.84
	corpus part	288.75 ± 6.39^a^	246.42 ± 8.29^b^	155.71 ± 3.68^c^	238.00 ± 5.16	249.00 ± 8.35	238.66 ± 1.65
	caudal part	342.22 ± 10.89^a^	288.40 ± 5.55^b^	199.59 ± 3.54^c^	260.67 ± 2.90	256.76 ± 4.79	249.45 ± 5.53
No. of germ cells (cells/1000 µm^2^)							
	spermatogonia	1.51 ± 0.07	1.29 ± 0.09	1.44 ± 0.09	1.34 ± 0.04	1.24 ± 0.05	1.38 ± 0.07
	spermatocytes	1.71 ± 0.07	1.74 ± 0.07	1.88 ± 0.1	1.66 ± 0.06	1.76 ± 0.04	1.61 ± 0.06
	spermatids	4.99 ± 0.15^a^	4.99 ± 0.24^a^	1.97 ± 0.13^b^	4.82 ± 0.15	4.94 ± 0.14	4.91 ± 0.15
Spermatozoal quantity (cells x 10^6^/g-testis)							
	daily sperm production	0.56 ± 0.02^a^	0.48 ± 0.02^b^	0.47 ± 0.01^b^	0.61 ± 0.01	0.62 ± 0.03	0.62 ± 0.02
	caudal epididymal spermatozoa	343.39 ± 12.70^a^	278.19 ± 13.98^b^	268.60 ± 11.24^b^	364.23 ± 16.31	357.39 ± 13.29	370.62 ± 17.31
Spermatozoal quality (%)							
	spermatozoal motility	76.21 ± 1.46^a^	65.15 ± 2.43^b^	61.01 ± 4.35^b^	75.81 ± 2.47	75.72 ± 1.59	73.38 ± 2.72
	spermatozoal viability	65.00 ± 2.27^a^	41.84 ± 1.72^b^	34.76 ± 3.26^b^	69.05 ± 2.36	69.94 ± 2.31	72.83 ± 1.63
	normal spermatozoa	46.14 ± 3.08^a^	32.92 ± 4.34^b^	31.29 ± 3.42^b^	49.79 ± 3.28	45.68 ± 3.49	40.76 ± 2.75
Types of abnormal spermatozoa (%)							
	tailless	9.12 ± 2.06	17.74 ± 7.07	16.18 ± 3.10	9.44 ± 0.88	9.79 ± 0.69	11.27 ± 1.68
	no hook head	8.19 ± 2.79	9.05 ± 1.99	8.85 ± 1.64	8.73 ± 2.40	8.79 ± 1.10	7.74± 2.65
	amorphous head	0.52 ± 0.14	1.01 ± 0.28	1.42 ± 0.56	0.24 ± 0.04	0.54 ± 0.32	0.68 ± 0.38
	neck abnormal	2.02 ± 0.42	2.83 ± 0.54	2.51 ± 0.54	1.33 ± 0.31	1.59 ± 0.54	1.97 ± 0.29
	tail abnormal	33.07±3.01	36.514.36	38.92±3.15	27.813.90	29.152.30	29.51±4.13
	tailless	9.12 ± 2.06	17.74 ± 7.07	16.18 ± 3.10	9.44 ± 0.88	9.79 ± 0.69	11.27 ± 1.68
Acrosomal intactness (%)		90.43 ± 0.77	88.88 ± 1.15	84.50 ± 2.39	90.73 ± 0.77	89.27 ± 0.55	88.69 ± 1.78
Acrosome-membrane intactness (%)							
	acrosome-membrane intact	41.47 ± 6.01	23.42 ± 3.87	20.18 ± 3.02	35.94 ± 7.43	36.55 ± 7.08	41.94 ± 7.37
	acrosome damage- membrane intact	41.76 ± 4.23	49.88 ± 3.73	51.65 ± 3.13	29.20 ± 5.72	29.73 ± 2.59	35.28 ± 2.55
	acrosome intact-membrane damage	0.05 ± 0.01	0.05 ± 0.02	0.06 ± 0.03	0.00 ± 0.00	0.00 ± 0.00	0.00 ± 0.00
	acrosome-membrane damage	16.62 ± 3.93	26.70 ± 4.59	28.11 ± 2.81	16.37 ± 4.26	19.05 ± 5.36	14.88 ± 3.40

One-way ANOVA and least significant difference (LSD) were employed.

Values in the same row not shown by common letters (a, b and c) were significant difference at p<0.05.

† Results are expressed as the mean±SD.

## Discussion

The *M. charantia* seed extracts at 400 and 800 mg dry matter/kg body weight didn’t affect changes in body weight and accessory reproductive organ weight but reduced diameter of seminiferous tubules and epididymides of male Wistar rats. However, *M. charantia* ethanol seed extracts provided to male Albino rats via the esophageal and peritoneal routes at 25 mg/100 g body weight for 35 days significantly reduced the weight of testes, epididymides, prostate glands and seminal vesicles, and the diameter of seminiferous tubules except body weight as well as *Albizza lebbeck* methanolic pod extracts at 50, 100 and 200 mg dry matter/kg body weight and *Amalakyadi churna* fruit extracts at 400 mg dry matter/kg body weight (5, 15, 16). 

*Albizza lebbeck* methanolic pod extracts and *Amalakyadi churna* fruit extracts also significantly reduced diameter of epididymides. *M. charantia* fruit extracts at 500 and 800 mg dry matter/kg body weight given to Wistar rats for 14 days didn’t cause the change in body weight (9, 15, 16). This study firstly demonstrated an infertility action of the extracts in male Wistar rats. Dehghan *et al* concluded that 100 mg/kg body weight of Iranian neem seed alcoholic extract caused a significant reduction in fertility which can be due to the decrease in caudal epididymal sperm motility and their morphological abnormalities (2). Naseem *et al *focused merely on an action of spermatogenesis arrest in male Albino rats given *M. charantia* ethanol seed extracts at 25 mg/100 g body weight (5). *M. charantia* stem extracts had an adverse effect on fertility in dog (17). 

The withdrawal of *M. charantia* ethanol seed extracts firstly revealed the temporal infertility in male Wistar rats. *Carica papaya* chloroform seed extracts and *Melia azadrach* seed oil also demonstrated temporal male infertility. Tumkiratiwong and Lerkchundhakriat reported that a combined pyrimethamine-sulfanilamide at 25 and 170 mg/kg body weight given to male Wistar rats for 49 days and followed by 35 days of drug withdrawal potentiated to the temporal infertility effect (18-20). The extracts at 400 and 800 mg dry matter/kg body weight significantly reduced the number of caudal epididymal spermatozoa. 

Lohiya *et al* provided *Carica papaya* chloroform seed extracts at 50 mg dry matter/kg body weight to langur monkey for 360 days and founded that on day 30 and 60, the number of caudal epididymal spermatozoa significantly reduced and on day 90, 120, 150, 180, 210, 240, 270, 300, 330 and 360, the sperm are totally death, but following 150 days of the withdrawal period, the number and viability of sperm were returned to normal (18). Parandin *et al* reported that *Melia azadrach* seed oil at 50 and 100 mg dry matter/kg body weight provided to rats for 60 days lowered live spermatozoa and following 90 days of the withdrawal period, the number of spermatozoa were returned to normal (19).

The extracts at 400 and 800 mg dry matter/kg body weight lowered daily sperm production, reflecting to the reduction in caudal epididymal sperm count. Dehghan *et al* reported that 100 mg/kg body weight of Iranian neem seed alcoholic extract decreased caudal epididymal sperm motility (2). *M. charantia* seed extracts at 25 mg/100 g body weight given for 35 days to male Albino rats reduced caudal epididymal spermatozoa (5). A reduction in caudal epididymal spermatozoa might be attributed to an occlusion at site of rete testis (21). 

The extracts significantly lowered percent of motile sperm. The motility of spermatozoa was completely established in epididymis. Consequently, the damages to caput, corpus and caudal parts of the epididymis effected sperm motility. Normal morphology of human spermatozoa is correlated to fertility and pregnancy rate both in vitro and in vivo (22, 23). The process of change in human sperm morphology is occurred in both seminiferous tubules and epididymides (24). This study showed a significant reduction in normal sperm morphology following providing the extracts at 400 and 800 mg dry matter/kg body weight to male Wistar rats, reflecting to the impairment of seminiferous tubule and epididymal malfunction leading to infertility. Shetty and Narayana reported that the abnormal sperm morphology could not pass through oviduct and/or fertilize the ovum in Wistar rats (25). The extracts at 400 and 800 mg dry matter/kg body weight caused a significant reduction in percent of acrosome-membrane intactness. It was reported that gossypol damaged acrosome of spermatozoa which leading to infertility in chicken (26). 

This study found that acrosome and cell membrane were can damage. The extracts at 400 and 800 mg dry matter/kg body weight leaded to the significant atrophy of seminiferous tubule as a consequence of a significant reduction in the diameter of seminiferous tubules. Density of spermatids, except of spermatogonia and of spermatocytes, was lowered in seminiferous tubules of the rats treated with the extracts at only 800 mg dry matter/kg body weight. According to Naseem *et al* *M. charantia* ethanol seed extracts at 25 mg/100 g body weight given to male Albino rats for 35 days reduced the number of spermatocytes, spermatids and spermatozoa except spermatogonia (5). *Carica papaya* chloroform seed extracts at 50 mg dry matter/kg body weight providing to langur monkey for 360 days had no effect on the number of spermatogonia (18).

Pyknosis nucleus was appeared in the cells of seminiferous tubules of Wistar rats treated with the extracts at 400 and 800 mg dry matter/kg body weight and the multinucleated giant cell was exist in lumen of seminiferous tubules of Wistar rats provided the extracts at only 800 mg dry matter/kg body weight. Multinucleated giant cells in seminiferous tubules were observed in seminiferous tubules of male Wistar rats treated with a combined pyrimethamine-sulfanilamide at 25 and 170 mg/kg body weight for 49 days (20). Pyknosis nucleus is caused by irreversible cell injury by which chromatin inside nucleus was permanently contracted into a smaller size and indefinitely apoptosis and multinucleated giant cell is characterized by the chronic inflammation of tissues caused by an aggregation of macrophages which incompletely engulfed foreign substances (27).

The extracts at 400 and 800 mg dry matter/kg body weight caused atrophy of epididymides and epididymal spermatozoa reduction in three portions of epididymis but the presence of plasma cells was found only in the corpus and caudal parts of epididymis. The multinucleated giant cells were exist only in the caput part of epididymis in male Wistar rats treated with the extracts at only 800 mg dry matter/kg body weight. Plasma cells are characterized by larger than leukocytes and quite oval nucleus (27). Multinucleated giant cells were found in lumen of seminiferous tubules of azoospermia man and it was either a mechanism of eradication of the death spermatozoa by phagocytosis or a mechanism of autolysis of spermatozoa (28).

We suggest that pathological changes in seminiferous tubules and epididymides disturbed testicular and epididymal functions leading to the reduction in quality and quantity of spermatozoa, including daily sperm production and caudal epididymal spermatozoa, the percentage of motile sperm, alive sperm, normal sperm morphology and acrosome-membrane intactness which all caused infertility. There were many reports regarding on infertility related to reduction in quantity and quality of spermatozoa and testicular and epididymal damages. *Bougainvillea spectabilis* aqueous leave extracts decreased the number of spermatozoa and atrophied seminiferous tubules in Swiss Albino mice (29). 

An active ingredient, triptolide, of *Tripterygium wilfordii* stem extracts lowered the number of seminal and motile spermatozoa (30). *Citrullus colocynthis* ethanol fruit extracts and *Cissampelos pareira* aqueous leave extracts at 1.0 ml/100 g body weight given for 14 days reduced seminal spermatozoa and increased the percentage of abnormal spermatozoa in Albino rats (31, 32). Gossypol inhibited spermatogenesis (33). Twenty and eighty percent of necrozoospermia in man were caused by defects of epididymis per se, and of both seminiferous tubules and epididymides, respectively (34). In this study, following 14 days of the withdrawal of the extracts, infertility became normal, corresponding well to a recovery of the damaged seminiferous tubules and epididymides which in turn daily sperm production, caudal epididymal spermatozoa, the percentage of motile sperm, alive sperm, normal sperm morphology and acrosome-membrane intactness were restored to normal. 

This study demonstrated that the level of seminal testosterone was higher than that of plasma testosterone 50 folds. Naseem *et al* reported that *M. charantia* seed extracts inhibited steroidogenesis by which large amounts of cholesterol were accumulated in steroidogenic cells and in turn might not be converted it into testosterone (5). The aqueous leaf extract of *Momordica charantia* at 12.5, 25.0 and 50.0 g of the powdered specimens with no mention of daily dose of the extracts induced the reduction in plasma FSH and testosterone levels in a dose dependent manner in adult male Wistar rats (35). The seminal testosterone level of 50-70 ng/ml is crucial for spermatogenesis in rats (36). The reduction to 2/3 of normal seminal testosterone level (approximate 33-47 ng/ml) didn’t have a direct adverse effect on spermatogenesis but more reduction below such a ratio would arrest spermatogenesis (37). 

The extracts at 400 and 800 mg dry matter/kg body weight lowered the seminal testosterone level less than the normal level and belowed such a level mentioned above. There was no any report concerning the direct disturbance of *M. charantia* ethanol seed extracts on secretions of hypothalamic gonadotropin-releasing hormone (GnRH) and/or anterior pituitary gonadotropins. The reduced seminal and plasma testosterone in Wistar rats probably reflects a response of the hypothalamus-pituitary-gonad axis (HPG axis) to *M. charantia* ethanol seed extracts, especially 800 mg dry matter/kg body weight.

This study demonstrated that the reduction in quality and quantity of spermatozoa was partly attributed to the reduced seminal and plasma levels of testosterone. Creasy showed that the reduced spermatids is associated to apoptosis of germ cells as testosterone plays an essential role on development of spermatocytes and spermatid stages 7-8 and 9-14 (38). The atrophy of seminiferous tubules caused by the reduction in the number of germ cells and secretions in seminiferous tubules were correlated to the reduced testosterone (38). 

Dohle *et al* explained that seminal testosterone acts directly on Sertoli cells and the reduced testosterone suppressed the synthesis of gene-coded androgen receptors in Sertoli cells (39). Low testosterone level caused an incomplete spermatogenesis and consequently, apoptosis of Sertoli cells (39). In this study, the reduced testosterone levels might be causative to significant reductions in daily sperm production and caudal epididymal spermatozoa. Additionally, testosterone is also important for maturation of spermatozoa in epididymides (39, 40).

## Conclusion

In conclusion, our study showed that 400, especially, 800 mg dry matter/kg body weight/day of *M. charantia* ethanol seed extracts caused the infertility in male Wistar rats. The fertility was probably attributed to the direct toxic effect on seminiferous tubules and epididymis and reduction in seminal and plasma testosterone levels which might significantly impacts on sperm motility, live spermatozoa, normal morphology of spermatozoa and acrosome-membrane intactness.

## Footnote

***This article extracted from M.Sc. thesis. (Ravicha Ploypattarapinyo)***

## References

[1] Mosher WD, Pratt WF (1991). Fecundity and infertility in the United States: incidence and trends. J Fertil Steril.

[2] Dehghan MH, Martin T, Dehghanan R (2005). Antifertility effect of Iranian neem seed alcoholic extract on epididymal sperm of mice. Iran J Reprod Med.

[3] Rastogi RP, Mehrotra BN (1993). Compendium of Indian Medicinal plants.

[4] Scartezzini P, Speroni E (2000). Review on some plants of Indian traditional medicine with antioxidant activity. J Ethnopharmacol.

[5] Naseem MZ, Patil SR, Ravindra SR, Patil RS (1998). Antispermatogenic and androgenic activities of Momordica charantia (Karela) in albino rats. J Ethnopharmacol.

[6] Patil SA, Patil SB (2011). Toxicological studies of Momordica charantia Linn. Seed extracts in male mice. Int J Morphol.

[7] Yama OE, Osinubi AA, Duru FIO, Noronha CC, Okanlawon AO (2011). Contraceptive effect of methanolic extract of Momordica charantia seed in male Sprague Dawley rats. Asian J Pharm Clin Res.

[8] Sharanabasappa A, Vijaykumar B, Saraswati BP (2002). Effect of Momordica charantia seed extracts on ovarian and uterine activities in Albino rats. Pharm Biol.

[9] Abalaka ME, Olonitola OS, Onaolapo JA, Inabo HI (2009). Evaluation of acute toxicity of Momordica charantia extract using Wistar Rats to determine safety levels and usefulness of the plant in ethnochemotherapy. Int J P App Sci.

[10] World Health Organization (1999). WHO Laboratory Manual for the Examination of Human Semen and Sperm-Cervical Mucus Interaction.

[11] Amann RP, Howards SS (1980). Daily spermatozoal production and epididymal spermatozoal reserves of the human male. J Urol.

[12] Luna LG (1968). Manual of Histological Staining Methods of the Armed Force Institute of Pathology.

[13] Linares V, Albina ML, Belles M, Mayayo E, Sanchez DJ, Domingo JL (2005). Combined action of uranium and stress in the rat. II. Effects on male reproduction. Toxicol Lett.

[14] Abul HT, Mathew TC, Abul F, Al-Sayer H, Dashti HM (2002). Antioxidant enzyme level in the testes of cirrhotic rats. Nutrition.

[15] Gupta RS, Kachhawa JB, Chaudhary R (2004). Antifertility effects of methanolic pod extract of Albizzia lebbeck (L.) Benth in male rats. Asian J Androl.

[16] Seetharam YN, Sujeeth H, Jyothishwaran G, Barad A, Sharanabasappa G, Umareddy B, Vijaykumar MB, Patil SB (2003). Antifertility effect of ethanolic extract of Amalakyadi churna in male Albino mice. Asian J Androl.

[17] Bhargava SK (1988). Antifertility agents from plants. Fitoterapia.

[18] Lohiya NK, Manivannan B, Mishra PK, Pathak N, Sriram S, Bhande SS, Panneerdoss S (2002). Chloroform extract of Carica papaya seeds induces long-term reversible azoospermia in langur monkey. Asian J Androl.

[19] Parandin R, Sadeghipour HR, Haeri Rohani SA (2008). Evaluation of antifertility effect and recovery of the seed oil constituents of Iranian species of Melia azadrach L. in male rats. J Reprod Contracept.

[20] Tumkiratiwong P, Lerkchundhakriat K (2011). Effect of a pyrimethamine-sulfanilamide combination on induced temporal infertility in male Wistar Rats. Kasetsart J (Nat Sci).

[21] Goyal HO, Braden TD, Mansour M, Williams CS, Kamaleldin A, Srivastava KK (2001). Diethylstilbestrol-treated adult rats with altered epididymal sperm numbers and sperm motility parameters, but without alterations in sperm production and sperm morphology. Biol Reprod.

[22] Garrett C, Liu DY, Clarke GN, Rushford DD, Baker HW (2003). Automated semen analysis: zona pellucida preferred sperm morphometry and straight-line velocity are related to pregnancy rate in subfertile couples. Hum Reprod.

[23] Liu DY, Garrett C, Baker HW (2003). Low proportions of sperm can bind to the zona pellucida of human oocytes. Hum Reprod.

[24] Auger J (2010). Assessing human sperm morphology: top models, underdogs or biometrics?. Asian J Androl.

[25] Shetty AJ, Narayana K (2007). The effects of carbamazepine on sperm morphology in Wistar rats. Indian J Physiol Pharmacol.

[26] Mohan J, Panda JN, Singh US, Moudgal RP (1989). Studies on antifertility effects of gossypol acetic acid in domestic cocks. J Reprod Fertil.

[27] Damjanov I (2005). High-Yield Pathology.

[28] Phadke AM, Phadke GM (1961). Occurrence of macrophage cells in the semen and in the epididymis in case of male infertility. J Reprod Fertil.

[29] Mishra N, Joshi S, Tandon VL, Munjal A (2009). Evaluation of anti-fertility potential of aqueous extract of Bougainvillea spectabilis leaves in Swiss Albino mice. IJPSDR.

[30] Hikim AP, Lue YH, Wang C, Reutrakul V, Sangsuwan R, Swerdloff RS (2000). Posttesticular antifertility action of triptolide in the male rat: evidence for severe impairment of cauda epididymal sperm ultrastructure. J Androl.

[31] Chaturvedi M, Mali PC, Ansari AS (2003). Induction of reversible antifertility with a crude ethanol extract of Citrullus colocynthis Schrad fruit in male rats. Pharmacology.

[32] Luangpirom A, Sirisarn W, Pontaisog J (2010). Antifertility activity of the aqueous leaf extract of Cissampelos pareira in male Albino mice. ABAH Bioflux.

[33] Frayne J, Hall L (1999). The potential use of sperm antigen as targets for immunocontraception: past, present and future. J Reprod Immunol.

[34] de Kretser DM, Huidobro C, Southwick GJ, Temple-Smith PD (1998). The role of the epididymis in human infertility. J Reprod Fertil.

[35] Odusoga AO, Ifabunmi OO, Ayokunle O (2014). Evaluation of oral administration of aqueous leaf extract of Momordica charantia on fertility hormones of adult male Wistar rats. Glob J Pharmacol.

[36] Turner TT, Jones CE, Howards SS, Ewing LL, Zegeye B, Gunsalus GL (1984). On the androgen microenvironment of maturing spermatozoa. Endocrinology.

[37] Zirkin BR, Santulli R, Awoniyi CA, Ewing LL (1989). Maintenance of advanced spermatogenic cells in the adult rat testis: quantitative relationship to testosterone concentration within the testis. Endocrinology.

[38] Creasy DM (2001). Pathogenesis of male reproductive toxicity. Toxicol Pathol.

[39] Dohle GR, Smit M, Weber RFA (2003). Androgen and male fertility. World J Urol.

[40] Cooper TG, Nieschlag E, Habenicbt UF (1992). The epididymis as a site of contraceptive attack. Spermatogenesis, fertilization and contraception.

